# Editorial Chit-Chat

**Published:** 1871-10

**Authors:** 


### EDITORIAL CHIT-CHAT.

DISGUSTED.—We have read Theodore Tilton's biography of Victoria C. Woodhull, and have been looking, ever since, for some friendly boot to kick us for the waste of time.

A SKILLFUL BUILDER.—Mr. Wyckoff's residence, when completed, will be the handsomest one in our city. It is a beauty, tastily built and ornamented by Mr. H. A. Plum, who is doing the finest work we have yet seen, anywhere in this vicinity.

MAKING LIGHT OF THE ANCIENT DEAD.—We see it stated in a German paper, that the French engineers have found use for the mummies in the catacombs of Egypt. They convert them into gas, and find their "candle power" much increased by the bituminous wrappings with which they are surrounded.

A RESTING PLACE.—Dr. P. H. Hayes, one of our contributors, who has furnished our journal with several excellent articles, has a Home for invalids, on the hillside at Watkins, N. Y. The building is a large and comfortable one, within ten minutes walk of the famous Glen, and overlooking the beautiful Seneca Lake.

The Doctor has had a large experience in caring for chronic invalids, and his Home is one of the most delightful in the country. The Institution is open Winter and Summer, for the reception of persons afflicted with chronic diseases, or who require a resting place from their business.

BAD PRACTICE—that of placing the face in a basin of cold water, then opening the eyes so as to allow the water to come in contact with them, under the supposition that it is "strengthening to the eyes."

We have been called upon recently, to treat several cases of severe ophthalmia caused by this practice.

BALLOON VOYAGING.—This system of navigation is again becoming popular. Many experiments are being made, by numerous aëronauts throughout the country, who find little difficulty in finding persons to accompany them upon their voyages. The siege of Paris by the Germans, opened a new era in aërial navigation, and the contagion has spread to the United States. The Parisean aëronauts demonstrated the entire feasibility of using balloons as a means of communication between the besieged and the outside world. From the many ascensions made, it appears that not a single loss of life has occurred, demonstrating the fact that aërial navigation is no more dangerous or hazardous than an ocean trip, in a modern steam ship.

PLASTIC SLATE ROOFING.—We've tried it,—tried it repeatedly—in fact, are continually trying it, much to the sacrifice of time and patience. You may discover us almost any day, after a pleasant and refreshing shower, on our housetop, shingle in hand, smearing plastic slate over the cracks and crevices in our *new* roof.

The sport is exhilerating as well as jolly, to say nothing of its being sticky, likewise dauby. It makes one of the most peculiar of roofs,—too fine for a seive, yet too coarse for a filter. The rain, as it falls upon the white bed-clothing in our spare room, is strongly impregnated with tar. Tar being good for consumption, 'tis there we send our phthisical friends to lodge. The result is miraculous—it either kills or cures. Should the rain be a heavy one, the bed and patient becomes so saturated as to bring the case to a speedy termination—they are drowned. But upon a clear night, it becomes a real enjoyment to occupy this bed, in our spare room, and watch the twinkling of the stars through the plastic-slate-roof, while you are inhaling the sweet perfume of the gas tar. Yes, plastic slate is a success, and it is with a sorrowing heart that we are having it removed and its place supplied with old-fashioned covering, known as shingles.

ELEGANT.—One of the finest parades ever witnessed in this city, occurred a few days since, upon the occasion of ST. OMER'S COMMANDERY'S visitation to Baltimore. Previous to their departure they gave our citizens an opportunity of witnessing their military evolutions, upon our principal thoroughfares. The Sir Knights made an elegant display in their handsome regalias, while LaFrance's band, in their beautiful, new uniforms, lent additional splendor to the parade.

We are sure that no finer appearing Commandery, or better Band, will be seen in the National conclave at Baltimore.

---

SAD—VERY.—Our neighbor recently was in the possession and enioyment of a beautiful and melodious feline. He was musical, was Thomas; could reach the higher notes, as well as the housetops, without the least apparent effort.—His favorite chanting place was upon the verandah, in front of our chamber, and the time of rehersal, midnight. Thomas died suddenly, last night, while performing a *sta-cat-o* in the upper *register*. In the *heat* of his excitement he died. He couldn't C. sharp, but would B flat, therefore, was he buried by the children, to-day, with becoming ceremony. Thomas, we hope, will not enter another furnace in the spirit land.

At noon, we saw a head board placed at his grave, upon which our eldest son had written, in good, school-boy latin, the following: "*Rest quiet cat in peace.*

---

"CONDURANGO."—This is a name of a tree of unknown botanical spices, a native of the province of Laga, in Southern Ecuador. It was first brought to the notice of the Surgeon-General, at Washington, by the Representative of Ecuador, in this country, who stated it was given, in decoction, by a wife of a sufferer from cancer, with a view of poisoning him, in order to relieve him from his suffering. It is stated that the poison at once relieved the patient from all pain, and that instead of killing him, finally cured the cancer.

However, true or false this story may be, a chemical analysis conducted by the most skillful chemists, fail to discover any active principle, that will produce the marvelous effects ascribed to it; and repeated trials and experiments with it, by our best medical men, has proven the so-called "cancer cure" to be utterly inert and worthless.

ROSENZWEIG,—The New York abortionist, who murdered the young woman found in a trunk, checked for Chicago, testified that he had a diploma from an "Electic Medical College, in Philadelphia," and that he paid $40 for it.

This is the same College that is represented by a certain professor, (?) who is perambulating the country, conferring the degree of his college upon all who will pay him forty dollars for the same. He was through this part of the country recently, and we hear of two or three "doctors," in Tioga County, Pa., who purchased his diploma.

It must be a "high old college," with a wonderfully profound and learned lot of graduates.

---

A QUEER TUMOR.—A friend sends us a copy of the *Indianapolis Evening News*, in which we find a glowing account of the removal of an ovarian tumor, by some Chicago surgeon. The heading of the article referred to, runs as follows:—"REMOVAL OF AN OVARIAN TUMOR FROM A LADY'S STOMACH, WEIGHING 35 POUNDS!"

That lady should at once be secured for Barnum's Great Museum. She would attract more attention from scientific men than all the rest of the "great show" combined. We are positive that it would be the *only* instance on record, where the ovaries found lodgment in the *stomach.*

The article, provokingly, leaves us to guess whether the stomach was removed with the tumor, and if so, whether the lady has experienced any difficulty with her digestion since the operation.

---

A NEW UTERINE SUPPORTER.—We extract the following notice from the Phila. *Med. & Surg. Reporter*. The description will answer equally well for a lawn mower, or a minuture threshing machine. When will physicians cease concocting such abominable traps?:

"A Wisconsin physician has patented a uterine supporter, having a soft rubber stem, within which is placed a nut and also a screw bolt passing through the nut, the bolt terminating at both ends in hard rubber parts of the instrument, and being provided with a knob at one extremity, by turning which the screw is rotated and the soft rubber stem lengthened or shortened accordingly."

That physician ought to be placed under arrest, before he has an opportunity to apply that abominable contrivance.

CHLOROFORM AND ROBBERY.—We frequently find statements in newspapers of robberies committed, by means of chloroform, administered the victims.

We doubt whether a case ever occurred, of chloroform being administered to a person while asleep, without awakening him. People have queer ideas of the manner in which chloroform is administered. We have seen statements of chloroform being injected through a key-hole, into a room where a person lay sleeping, and insensibility so produced. It would require more than a gallon of the anæsthetic to produce any stupifying effect upon an inhabitant of an ordinary sized sleeping apartment, when applied in the manner described; and *then* even, he would be fully aroused by the coughing that would certainly be produced, the moment the chloroform reached his lungs.

We should look with great suspicion upon one who claims to have been robbed through the agency of chloroform.

---

HOW TO STOP IT.—Recent discoveries of deaths from abortion, in New York City, reveals the fact that thousands of unborn infants are slaughtered there, yearly. Several notorious abortionists have become immensely rich, from fees received from women who have sought their services. Strange to say, most of these women are married, and sought to destroy their innocent babes for the reason that the requirements of fashion will not allow the time that is necessary to care for children. Therefore, the aid of the abortionist is sought, and secured, in order to give the fashionable mother more time to attend parties and watering-places.

Occasionally the life of the mother is sacrificed, and then only does the public hear of the murders committed by these villainous operators, that are to be found in every city and village.

We know of but one way by which this wholesale slaughter of innocent babes can be prevented; and that is, to make the mother an equal partner in the murder, with the abortionist. Surely, she is equally guilty, for it is she who almost invariably employs the services of the murderer.

Let a law be passed, making it a criminal offense, for a woman to apply to any physician for an abortion, and hold the physician liable if he does not report to the authorities, the fact of a woman applying to him with such criminal intent. Let a guard be placed over these well-known slaughtering pens, in the larger cities, with instructions to arrest every woman found entering them. It is more than likely that a few public arrests of this character, would deter other fashionables from exposing themselves to the same risks.

This abortionist is not wholly to blame. He cannot prosecute his business unless he has *willing* patients. Let them suffer with him.

